# A Novel Method for Cryopreservation of Human Neutrophils for Phenotypic Analysis

**DOI:** 10.3390/biology15211951

**Published:** 2026-10-30

**Authors:** Sandra Chishimba, Raffi Gugasyan, Stephen J. Rogerson, James G. Beeson

**Affiliations:** 1 Burnet Institute, Melbourne, VIC 3004, Australia; sandra.chishimba@burnet.edu.au (S.C.); raffi.gugasyan@burnet.edu.au (R.G.); 2 Department of Medicine, Royal Melbourne Hospital, Faculty of Medicine, Dentistry and Health Sciences, University of Melbourne, Melbourne, VIC 3050, Australia; 3 Department of Immunology, Central Clinical School, Monash University, Melbourne, VIC 3004, Australia; 4 Department of Infectious Diseases, Peter Doherty Institute, University of Melbourne, Melbourne, VIC 3000, Australia; sroger@unimelb.edu.au; 5 School of Translational Medicine, Monash University, Melbourne, VIC 3004, Australia

**Keywords:** neutrophils, cryopreservation, immunophenotype, immunity, flow cytometry

## Abstract

**Background:** Neutrophils play an essential role in pathogen clearance and can also contribute to pathogenesis and inflammation. Phenotypic changes of neutrophils during infection or inflammation may be associated with their functional activity. However, investigation of neutrophil phenotypes and functions in clinical studies is challenging because of their short viability ex vivo and difficulties in effectively cryopreserving neutrophils for subsequent analyses. **Methods:** We isolated neutrophils from human peripheral blood and evaluated different approaches for cryopreservation. We examined labelling of cell surface receptors of interest, including CD64, CD32, CD16, CD54, CD66b, CD62L and CD14, on fresh versus cryopreserved neutrophils by flow cytometry. Trypan blue staining was used to assess cell viability and membrane integrity. **Results:** We developed a neutrophil cryopreservation protocol that preserved cell immunophenotypes for downstream evaluation. All cell surface receptors tested could be labelled and quantified using flow cytometry. There were some differences in levels of surface staining for some receptors when comparing fresh and cryopreserved cells, but these differences did not interfere with the immunophenotypic evaluation of neutrophils. **Discussion:** Overall, the data suggest that our neutrophil cryopreserving and thawing protocols can preserve neutrophil surface receptors relevant to functions and phenotypes and are methods suitable for use in clinical studies.

## Simple Summary

Neutrophils are immune cells that play an essential role in the clearance of infections and can also be involved in certain inflammatory diseases. Analysing changes in the properties, or phenotypes, of neutrophils during infection or inflammation is important for understanding disease processes and immunity to inform the development of new treatments, vaccines, and diagnostics. However, investigation of neutrophil phenotypes and functions in clinical studies is challenging because of their short viability in the laboratory and difficulties in effectively cryopreserving neutrophils for subsequent analyses. To address this, we developed a neutrophil cryopreservation protocol, using neutrophils isolated from human blood, that preserved important phenotypes and properties of neutrophils and subsequently enabled detailed investigations. There were some differences in comparing fresh and cryopreserved cells, which need to be considered in further analyses. Overall, our study suggests that our neutrophil cryopreserving and thawing protocols can preserve neutrophil phenotypes relevant to clinical studies.

## 1. Introduction

Neutrophils are a group of immune cells with varying phenotypes and functions [1,2,3,4]. They are among the first line of defence during infection and have a role in modulating immune responses [5,6,7]. Neutrophils possess various effector mechanisms associated with protective immunity against pathogens. These include antibody and non-antibody mediated phagocytosis [8,9,10,11,12,13,14,15], cytokine, chemokine and granule release, reactive oxygen species (ROS) production, and neutrophil extracellular traps (NETs) formation [2,16,17,18].

Studying neutrophils is important for understanding the development of disease and immunity during acute infection, since they are among the early cellular immune responses. One way to investigate disease progression and immune responses is by examining and quantifying neutrophil receptor changes [19]. Studies investigating neutrophils have demonstrated that changes in surface receptor expression during inflammation and/or infection may be correlated with enhanced effector functional activity [20,21]. Surface receptors relevant to protective immunity, also referred to as ‘activation receptors’, include Fcγ receptors (FcγR) FcγRI (CD64), FcγRIIa (CD32a) and FcγRIII (CD16) [2,22,23] involved in phagocytosis and cellular cytotoxicity as well as adhesive and migratory molecules CD66b (carcinoembryonic antigen-related cell adhesion molecule 8 (CEACAM8)), CD54 (intercellular adhesion molecule 1 (ICAM-1)) and CD62L (an L-selectin or SELL) [7,19,24,25,26,27].

Investigation of immunophenotypic changes on neutrophils is typically achieved by quantifying receptors and other surface proteins using flow cytometry. In many clinical studies, access to flow cytometric analysis on freshly collected neutrophils may not be possible due to lack of access to suitable facilities, such as occurs in many clinical settings, community-based studies, and low-income or resource-constrained settings. Therefore, to investigate phenotypes, the isolation of neutrophils from peripheral blood samples and cryopreservation for storage and/or shipment is necessary. Development of a granulocyte cryopreserving method has been challenging over the years due to cell fragility, short viability ex vivo and post-thaw reduced effector function [28,29]. Nevertheless, fixed leukocytes can be cryopreserved, enabling post-thaw differentiation of cell populations and measurement of activation receptors despite post-fixation morphological changes [30,31].

Although some methods for immunophenotyping neutrophils in cryopreserved whole blood samples exist, neutrophil phenotyping remains a challenge [30,32]. Limitations of using cryopreserved whole blood for neutrophil immunophenotyping include alteration in neutrophil size and granularity that promote overlap with other leukocytes as well as reduced or lost antibody recognition of surface receptors due to cryopreservation-induced changes in receptor configuration [30,32]. This underscores the necessity to use purified cells when assessing immunophenotypic changes. However, limited data exist on purified cryopreserved neutrophil immunophenotyping, likely because purification and freeze–thaw steps may activate myeloid cells and alter their surface marker expression [33,34]. This restricts the capacity to study neutrophils in many clinical settings.

To address this gap, we developed a cryopreservation and thawing method that would allow us to investigate purified neutrophil immunophenotypes after cryopreservation in areas where no flow cytometer is available on site. We defined immunophenotype preservation as the retention of surface receptor labelling that is detectable on fresh neutrophils.

Receptors of interest in our studies included the expression of FcγRs (CD64, CD32 and CD16) and receptors CD66b, CD54 and CD62L, since changes in expression of these receptors are associated with an immune response during infection and/or inflammation.

## 2. Methods

### 2.1. Ethical Approval and Consent to Participate

Research involving human participants, human material, or human data was performed in accordance with the Declaration of Helsinki. Ethics approval for collection of blood to isolate neutrophils was provided by the Human Research and Ethics Committee of Alfred Health, Australia (Protocol number HREC/497/11). This study was not a clinical trial. All participants gave written informed consent.

### 2.2. Consent to Publish Declarations

Consent to publish declarations is not applicable.

### 2.3. Neutrophil Isolation, Cryopreservation and Thawing and Staining

The step-by-step protocol of the method is provided in the Supplementary File and is published on protocols.io. DOI: dx.doi.org/10.17504/protocols.io.4r3l21723g1y/v1.

Briefly, peripheral blood samples were collected from healthy adult residents in Melbourne, Australia. Whole blood was collected from 11 consenting adult Melbourne resident donors in Australia, with an age range of 22 years to 53 years. The blood was collected in lithium heparin tubes (BD Vacutainer LH 170 I.U., REF 367526; BD, Plymouth, UK) between 9 a.m. and 10 a.m.

Neutrophils were isolated by negative selection within 2 h of whole blood collection using an EasySep™ Direct Human Neutrophil Isolation Kit (Cat. No. 19666; StemCell Technologies, Vancouver, BC, Canada) following the manufacturer’s instructions. Isolated neutrophils were cryopreserved at 1 × 10^7^ cells/mL in RPMI-1640 medium (with sodium bicarbonate and lacking L-glutamine) (Cat. No. R0883; Sigma-Aldrich, Gillingham, UK) containing 10% bovine serum albumin (BSA; BovoStar, cat. No. BSAS 1.0; Bovogen Biologicals, Keilor East, Australia) and 7.5% dimethyl sulfoxide (DMSO; cat. No. D2650; Sigma-Aldrich, Gillingham, UK). Cell suspensions were transferred into Nunc CryoTube 1.8 mL vials (Thermo Scientific, Waltham, MA, USA; Cat. No. 368632), placed in Corning CoolCell^TM^ LX Cell Freezing Containers (Corning Life Sciences, Tewksbury, MA, USA; Cat. No. 432003) and stored at −80 °C. Cells were removed from the freezer, placed on dry ice, then rapidly thawed in a 37 °C water bath with gentle agitation, until a small ice crystal remained. The cell suspension was immediately transferred to 10 mL of cold thawing buffer 10% BSA in 1 × Hank’s Balanced Salt Solution (HBSS) [without calcium chloride, magnesium chloride, or magnesium sulfate] (Cat. No. 14185-052, Life Technologies, Grand Island, NE, USA) in a 14 mL polystyrene tube and washed once. The cells were then fixed for 10 min with 2% paraformaldehyde (PFA; cat. No. C0042B; ProSciTech, Kirwan, Australia) in 1 × phosphate-buffered saline without calcium and magnesium (PBS; cat. No. 14200-075; Gibco, Grand Island, NE, USA).

Fresh or cryopreserved neutrophils at (1 × 10^5^) were stained for 30 min at 4 °C in FACS buffer (1% BSA in calcium- and magnesium-free HBSS) with anti-human antibodies against CD66b (PerCP/Cyanine5.5, clone G10F5), CD16 (Brilliant Violet (BV) 650, clone 3G8 in the initial gating strategy fresh and cryopreserved; BV421, 3G8 in subsequent fresh and cryopreserved cells assays), CD14 (BV 750, clone 63D3), CD62L (Alexa Fluor, clone DREG-56), CD64 (APC, clone 10.1), CD32 (PE/Cyanine7, clone FUN-2), and CD54 (PE, clone HA58), all obtained from BioLegend (San Diego, CA, USA) except CD16 BV421, 3G8 obtained from BD Horizon, Franklin Lakes, NJ, USA). Antibody dilutions and reaction volumes were kept identical across single-stained controls and fully stained samples, with 25 µL reaction volume in 96-well plates to maximize efficiency and maintain consistency. Viability dye, fluorescence-minus-one (FMO), brilliant stain buffer and Fc block were not used. Phenotyping data were acquired using a BD^®^ LSR II flow cytometer (BD Biosciences, San Jose, CA, USA) using consistent voltages across experiments within each sample type; however, voltage settings differed between fresh and cryopreserved samples.

### 2.4. Gating Analyses

**Figure 1:**
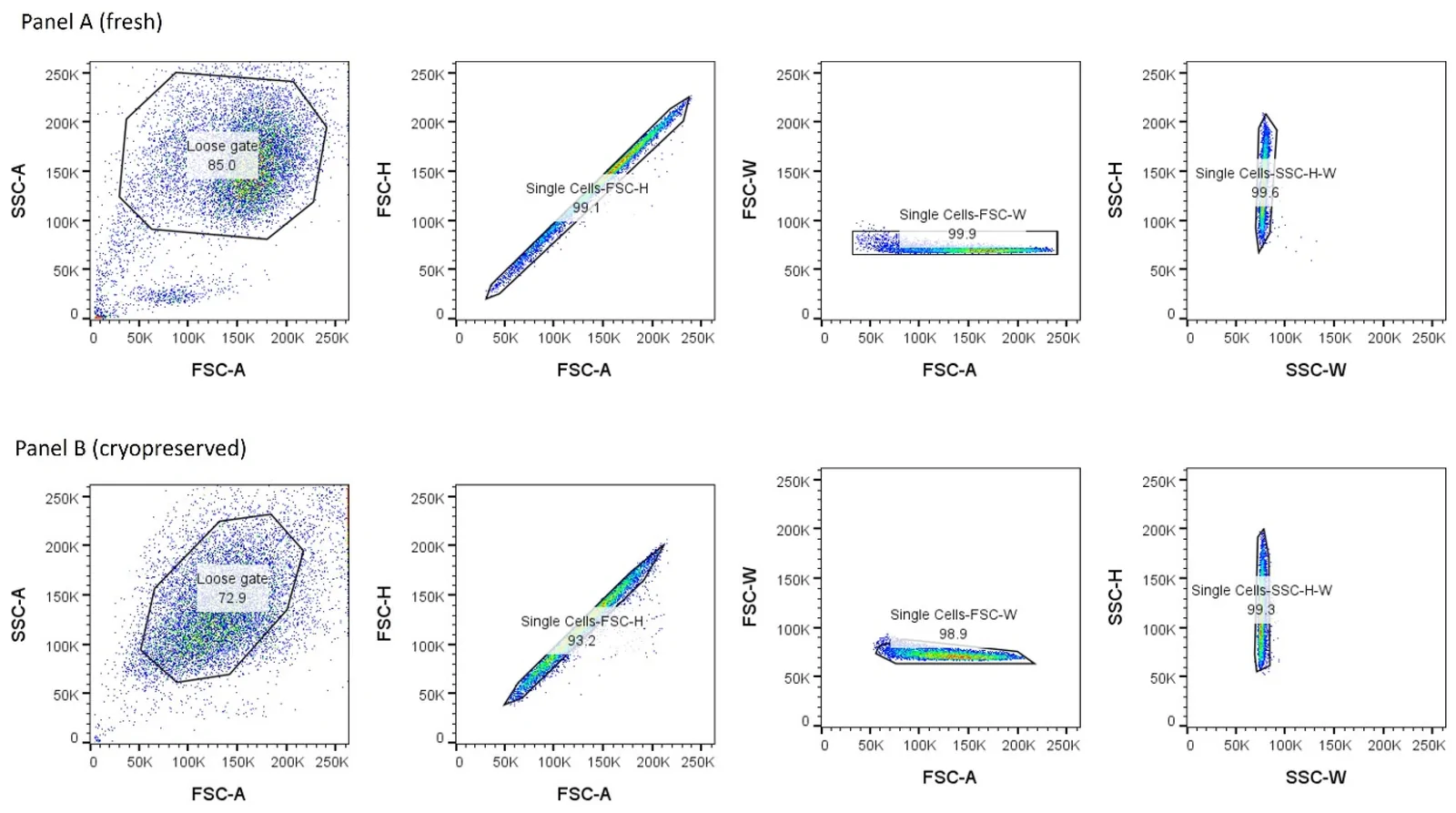
Flow cytometry hierarchy gating of fresh and cryopreserved neutrophils. Panel (**A**) represents fresh neutrophils; Panel (**B**) represents cryopreserved neutrophils. Fresh and cryopreserved neutrophils were isolated from the same donor on the same day. Fresh neutrophils were fixed after staining. Cryopreserved neutrophils were fixed after thawing and subsequently stained. Note, the SSC-A vs. FSC-A gate is referred to as a loose gate and not neutrophils—even though the neutrophil isolation kit can isolate neutrophils with up to 99% purity—for the purposes of sequential series of gating to exclude doublets and aggregates. All cells were fixed with 2% paraformaldehyde prior to analysis on the flow cytometer because of biosafety requirements. The fresh cell panel is a representative of four experiments, and the cryopreserved panel is a representative from seven experiments. Data analysed using FlowJo_v10.10.0_CL. Values indicate the percentage of cells that fall within the specified gate defined.

Flow cytometry data percentages, median fluorescence Intensity (MFI), stain index (SI), and dot plots were generated using FlowJo_v10.8.1 and FlowJo_v10.10.0_CL. Compensation matrices were calculated using FlowJo version 10.8.1 and 10.10.0_CL with unstained cells and single-stain controls. Neutrophils were used as the CD16 control, while compensation beads (BD CompBead Anti-Mouse Ig, κ/Negative Control Particles Set catalogue #BDB552843) were used for the remaining markers. We gated cells on SSC-A vs. FSC-A as phagocytes. Next, doublets were gated out by FSC-H vs. FSC-A, FSC width (FSC-W) vs. FSC-A, and SSC height (SSC-H) vs. SSC width (SSC-W) (Figure 1).

**Figure 2:**
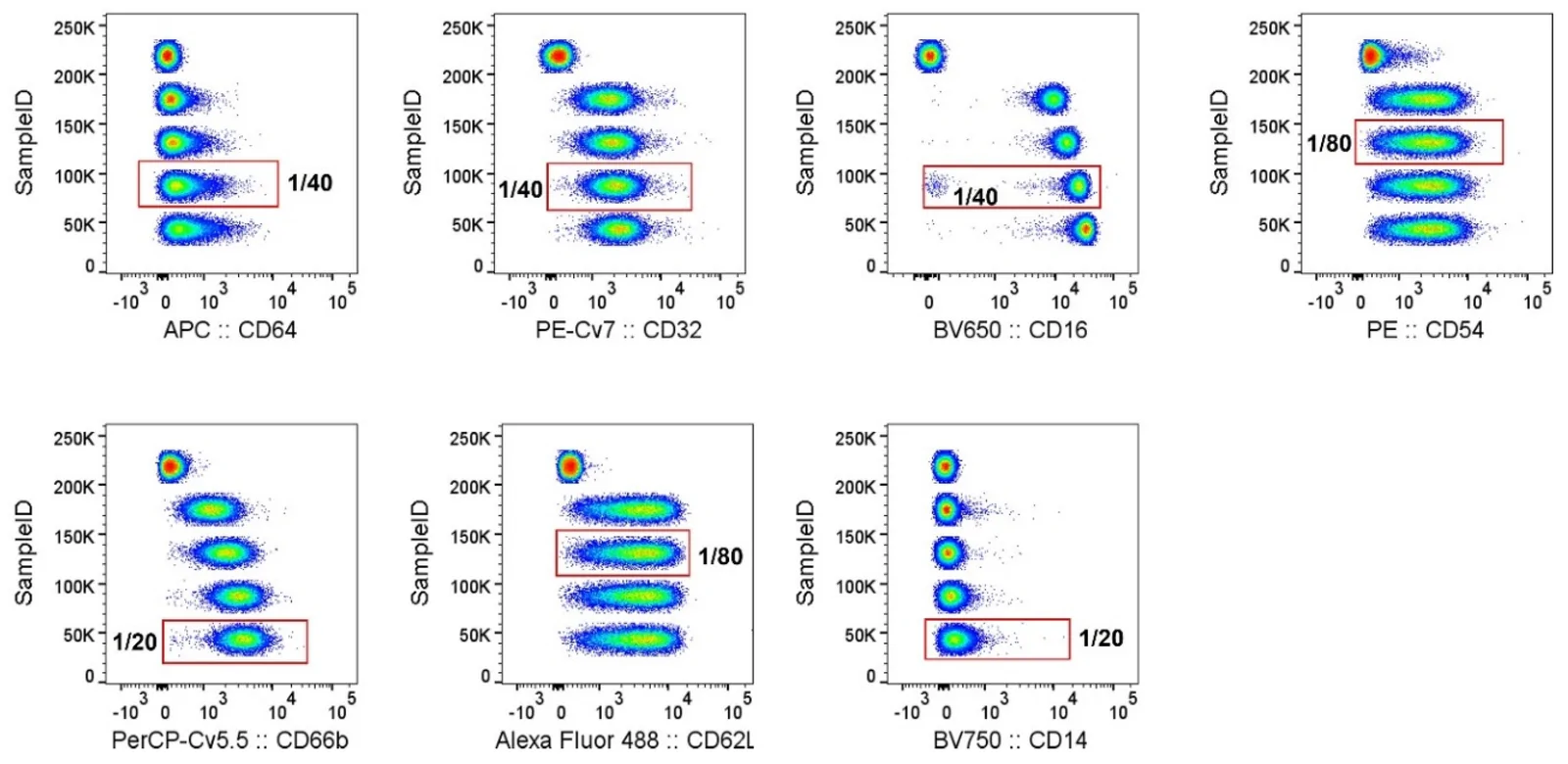
Antibody serial dilutions to label surface receptors of fresh neutrophils. Red highlighted rectangles on dot plots represent optimal concentrations identified and subsequently used in our flow cytometry assays for the specific markers. CD62L and CD54 were used at 1/80 dilution. CD64, CD32, and CD16 were used at 1/40 dilution. CD66b and CD14 were used at 1/20 dilution. The figures show a representative experiment out of two experiments. FlowJo_v10.8.1 was used to analyse receptor expression. Values on the *X*-axis are MFI.

Optimal antibody working concentrations were ascertained by titration of staining antibodies on freshly isolated neutrophils and a subset of cryopreserved neutrophils from freshly collected cells from the same donor. Fresh cells were fixed with 2% PFA after staining, while cryopreserved cells were fixed prior to staining. Positive and negative populations were gated using histograms, and antibody titrations quantified by SI. We finalized the antibody panel by concatenating the serial titrations and selecting the optimal single antibody dilutions based on the dilution that gives a good separation of positive and negative populations. The optimal antibody concentrations are highlighted in red (Figure 2).

**Figure 3:**
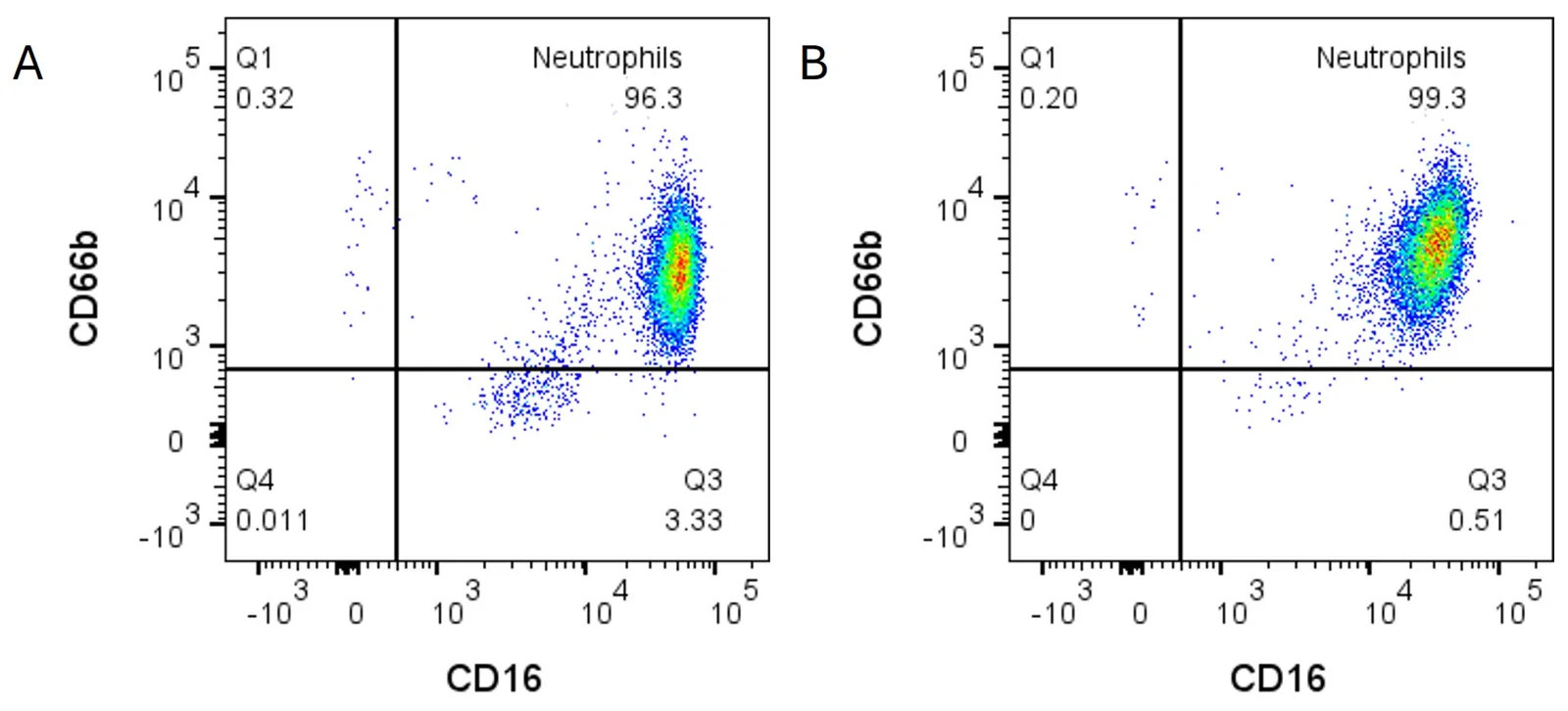
Neutrophil purity assessment (CD16^+^CD66b^+^) by flow cytometry. Neutrophils were isolated from whole blood using the EasySep™ Direct Human Neutrophil Isolation Kit (StemCell Technologies, Vancouver, BC, Canada; Cat. No. 19666). Neutrophil purity was assessed by flow cytometry using quadrant gating, with neutrophils identified within the CD16^+^CD66b^+^ gate. Images (**A**,**B**) show two isolations performed on different days using blood from the same donor. The images are representative of 7 independent experiments. Additional data are provided in Supplementary Figure S2.

Following antibody optimization, isolated neutrophils were further identified using CD16^+^CD66b^+^ gating, with boundaries defined using unstained and single-stained controls, to confirm purity and exclude non-neutrophil contaminants after isolation; purity ranged from 96.3% to 99.3% (Figure 3). We used a BD^®^ LSR II flow cytometer throughout these studies to reduce variation across experiments. Ten thousand events were acquired per sample on the SSC-H vs. SSC-W.

Descriptive statistics were used to report study findings, including the percentage of cells positive in flow cytometry for specified gating and MFI, and are indicated for each figure.

## 3. Results

### 3.1. Neutrophil Cryopreservation Protocol Validation

Several cryopreserving and thawing conditions were tested (see full list in Table 1 and examples shown in Figure S1a–c). Trypan Blue Stain (0.4%, Cat. No. T10282; Invitrogen, Life Technologies Corp., Eugene, OR, USA) exclusion was used as a measure of membrane integrity and viability with the different conditions that were tested. Trypan blue staining indicated high cell viability and membrane integrity when neutrophils were cryopreserved in 10% BSA + 7.5% DMSO in RPMI 1640 and then thawed and left resuspended in 10% BSA HBSS (Table 1 and Figure S2b). We compared cell viability based on the membrane integrity of fresh and cryopreserved neutrophils resuspended in 10% BSA HBSS. We found that cell viability based on membrane integrity for fresh cells ranged between 98 and 99.9% (Figure S1d), while cryopreserved cell viability ranged between 90 and 99.9% (Figure S1b). Cryopreserved neutrophil viability appeared to depend to some extent on neutrophil-to-suspension buffer (10% BSA HBSS) ratios after thawing. Specifically, pellets with a cell count of approximately 1 × 10^7^ cells/mL showed high cell membrane integrity when resuspended in 500 µL buffer. Cells with concentration more than 1 × 10^7^ cells/mL required resuspension in a medium range, between 600 µL and 1000 µL. Larger neutrophil pellets after thawing may require extra buffer to maximize cell viability based on membrane integrity.

Forty-five minutes to an hour after thawing, neutrophil cell viability and membrane integrity appeared compromised. Because cell integrity deteriorated after 45–60 min following thawing, cells were immediately fixed following thawing with 2% PFA final concentration. In contrast, fresh neutrophils were fixed after staining, which is the standard procedure used. Based on these studies, a step-by-step protocol was developed, which is provided in the Supplementary File.

### 3.2. Neutrophil Surface Receptors Are Preserved After Cryopreservation

We investigated whether neutrophil structural changes can occur following cryopreservation. We compared fresh and cryopreserved neutrophils. The fresh and cryopreserved neutrophils were obtained from the same Melbourne donor and isolated on the same day. Fresh and frozen cell acquisition was performed at different time points. We observed differences in the forward scatter area (FSC-A) versus (vs.) side scatter area (SSC-A) between fresh and cryopreserved neutrophils during flow cytometry cell acquisition, suggesting some changes in size and granularity (Figure 1). For that reason, we acquired fresh and cryopreserved neutrophil flow cytometry data using different FSC-A and SSC-A voltages.

**Figure 4:**
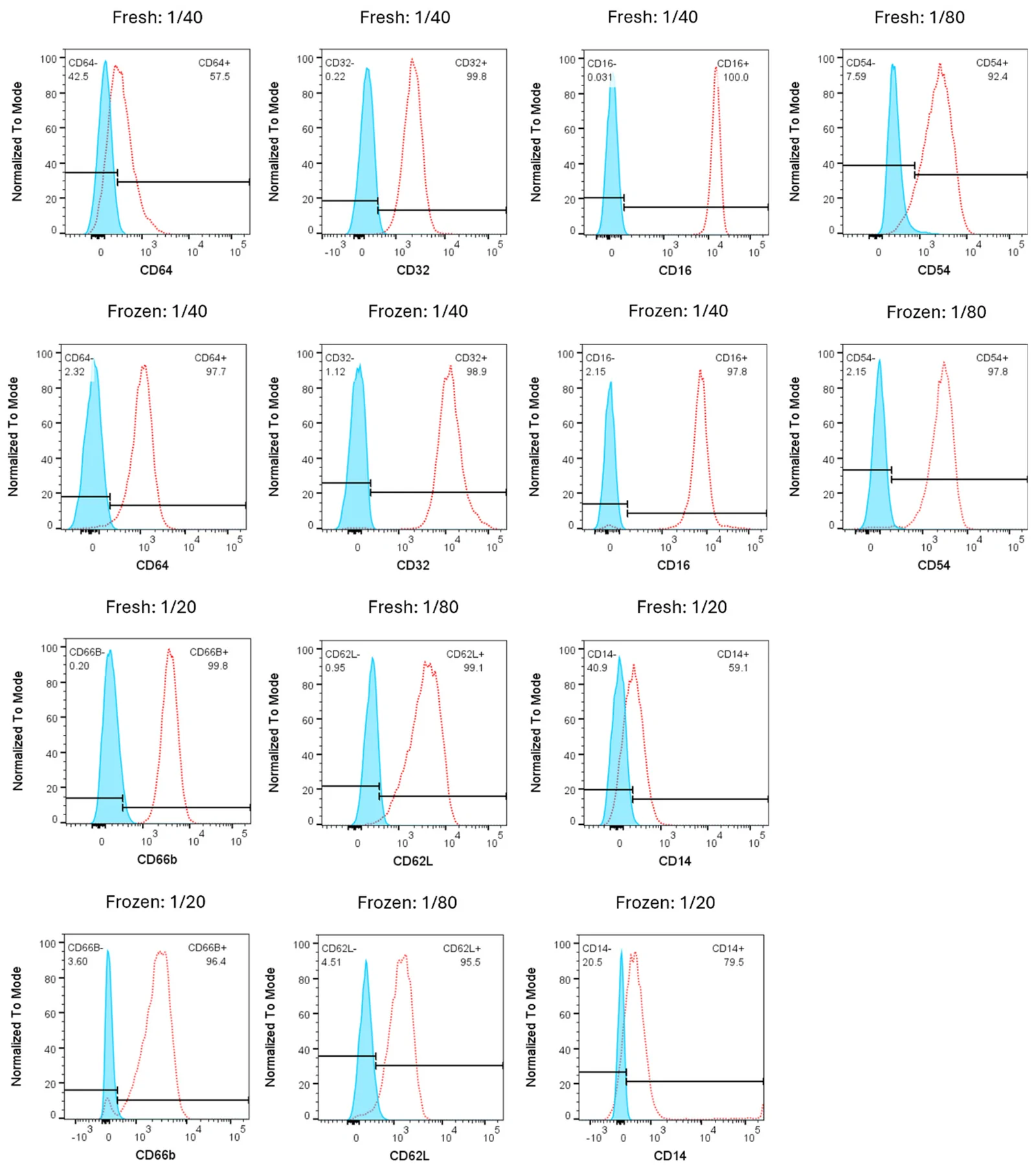
Comparison of the frequencies of receptor-positive, single-stained fresh and cryopreserved neutrophils at the respective optimal antibody dilutions. Top panel represents fresh cells, while bottom panel represents cryopreserved cells; antibody staining at optimal concentrations. Neutrophils were isolated from one donor. A subset was stained immediately after isolation, while the remaining neutrophils were cryopreserved and stained later. Light blue histograms represent unstained cells, while red dotted histograms show stained cells. Interval gates highlight the separation between the positive and negative populations. The graphs were designed in FlowJo_v10.8.1. The fresh cell panel is a representative of four experiments, and the cryopreserved panel is a representative of two experiments. Cells were stained for 30 min on ice. Data were analysed using FlowJo_v10.8.1. The percentage of cells within each gate are stated for each figure. Values on the *X*-axis are MFI.

We then examined whether a range of cell surface receptors of interest, including CD64, CD32, CD16, CD54, CD66b, CD62L and CD14, were labelled on cryopreserved neutrophils. These surface receptors were selected on the basis that they are important in neutrophil functions relevant to immunity to infectious pathogens. We compared the optimized staining of our target receptors on fresh neutrophils versus cryopreserved neutrophils from the same donor isolated on the same day. Our data show that all markers detected on fresh cells were also detectable on cryopreserved neutrophils (Figure 4). The proportions of cells expressing CD64 and CD14 were somewhat higher in cryopreserved cells compared to fresh cells, but the cell surface molecules could be clearly labelled in fresh and cryopreserved neutrophils (Figure 4). Data from testing additional donors are provided in the Supplementary File (Figure S2A–D); for each donor sample, flow cytometry data show cell gating and labelling with antibodies to specific cellular receptors.

**Figure 5:**
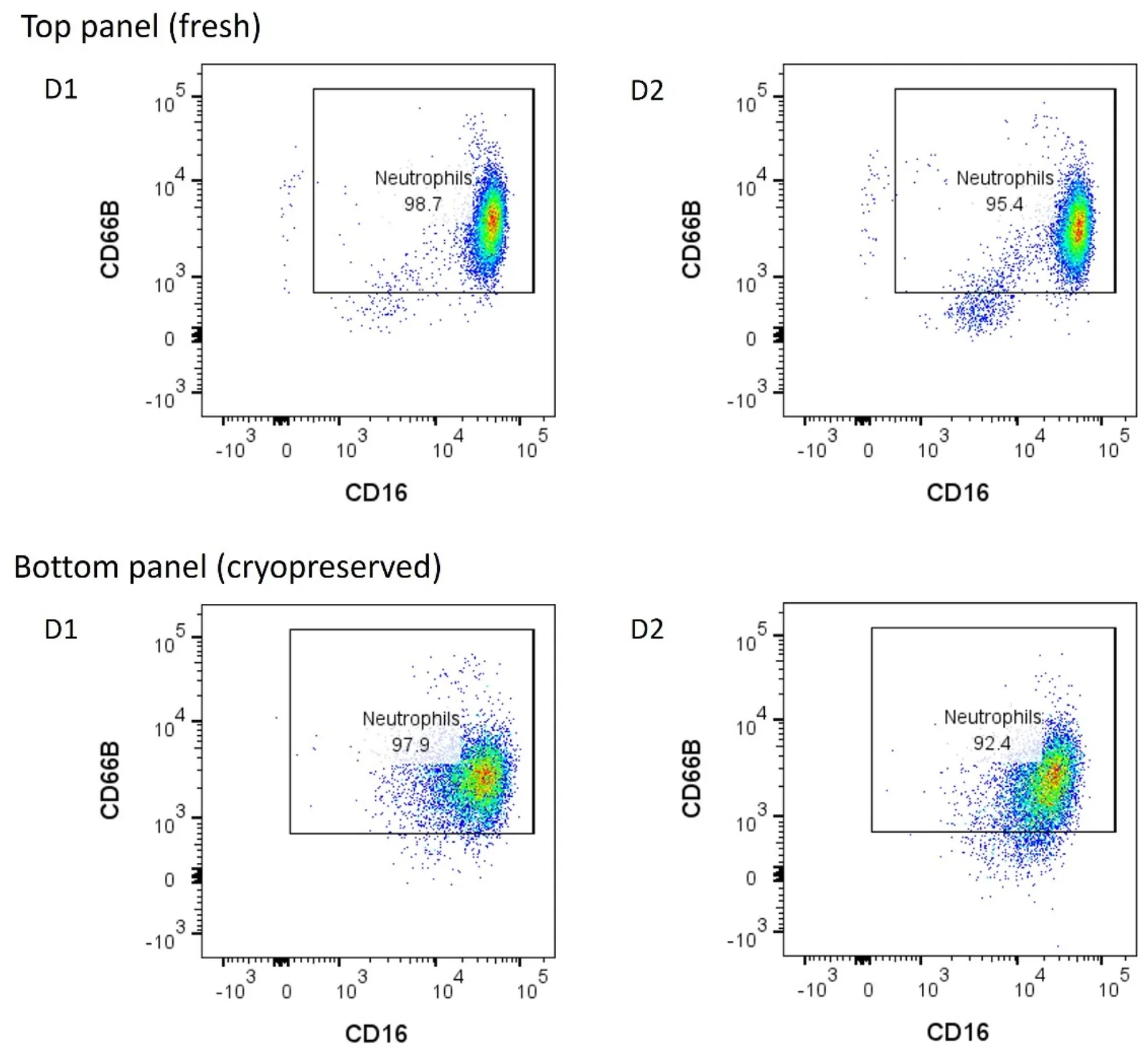
Fresh and cryopreserved neutrophil population gating. Top Panel, fresh cell gating on neutrophils from two biological replicates: Donor 1 (D1) and Donor 2 (D2). Bottom Panel, cryopreserved cell gating on neutrophils from two biological replicates (D1 and D2). Neutrophils were defined by CD16^+^CD66b^+^ rectangular gates. A subset of neutrophils isolated from two donors were cryopreserved and later run in duplicate. Data were analysed using FlowJo_v10.10.0_CL. The percentage of cells within each gate are stated for each figure. Values on the *X*-axis and *Y*-axis are MFI. Data from additional donors are provided in Supplementary Material Figure S2.

We next performed multi-colour flow cytometry to evaluate surface receptors of interest on both fresh and cryopreserved neutrophils. Compensation beads were used to ensure signal accuracy. We gated our target cells on high FSC-A and high SSC-A. Next, doublets were excluded by FSC-H vs. FSC-A, FSC-W vs. FSC-A, and SSC-H vs. SSC-W gates (Figure 1). Subsequently, neutrophils were gated on CD16 vs. CD66b (Figure 5). Neutrophil purity within the CD16^+^CD66b^+^ gate ranged from 96.8% to 99.3%. We observed minor differences in cell-surface fluorescence intensity distribution between cryopreserved and fresh neutrophils within the CD16^+^CD66b^+^ gate. Specifically, a small CD16^dim^CD66b^dim^ neutrophil population was detected in the fresh preparation but absent following cryopreservation. Despite these differences, the proportion of gated cells was similar between fresh and cryopreserved samples (Figure 5: top panel (fresh) and bottom panel (cryopreserved); Supplementary Material, Figure S2). Sparse, non-clustered populations of CD66b^+^ only and CD16^+^ only cells were also observed.

**Figure 6:**
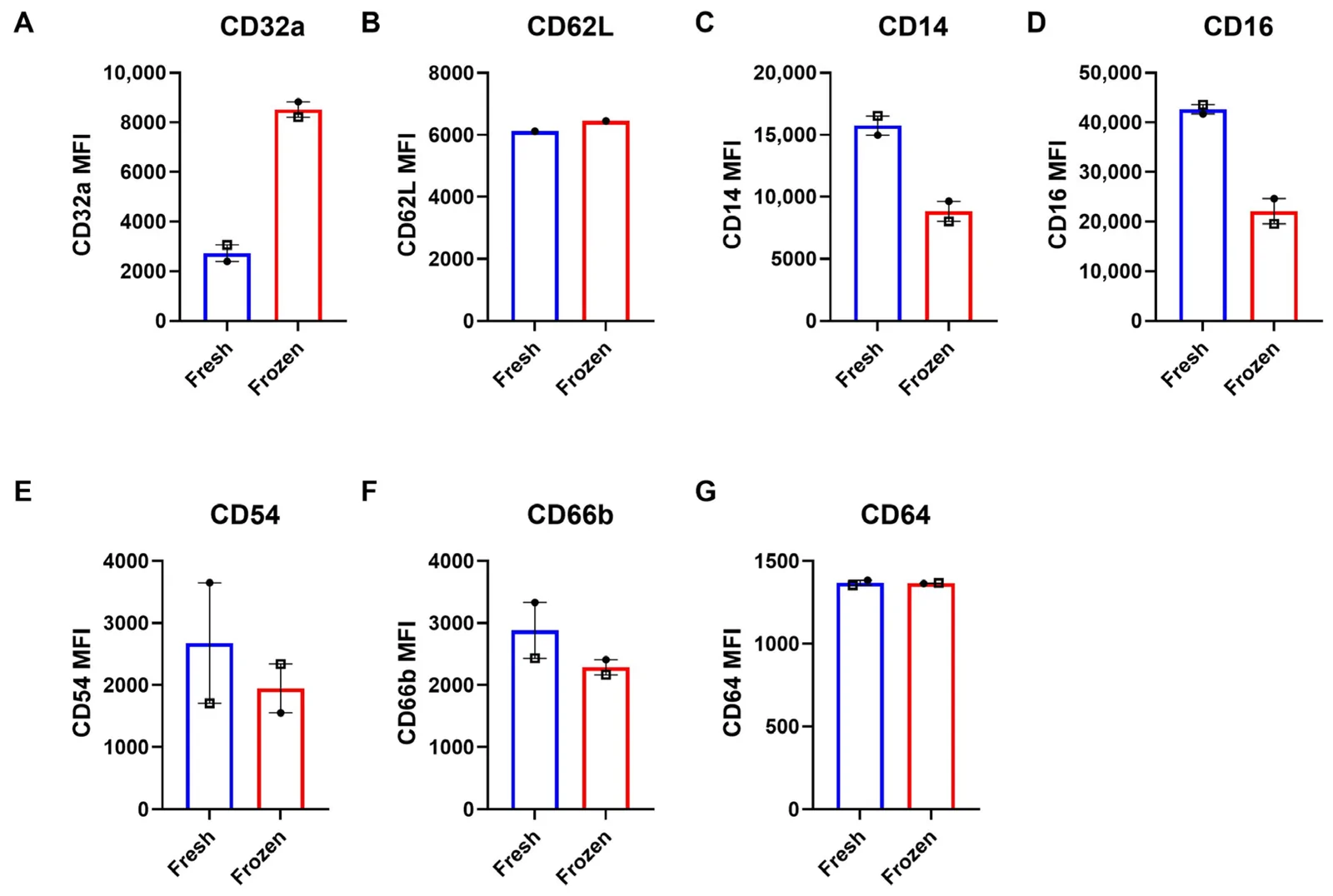
Multi-colour flow cytometry profile of surface receptors on fresh versus cryopreserved neutrophils. This figure shows receptor labelling on neutrophils from two donors (tested in duplicate) from representative experiments. Receptors on fresh and frozen neutrophils were quantified by mean fluorescence intensity (MFI). Neutrophils were isolated from two donors, and a subset of cells was cryopreserved per donor. Samples were run in duplicate. In the bar graph, dots/circles represent donor 1 and squares represent donor 2. Each dot point or square represents the average of technical replicates. (**A**) CD32 expression, (**B**) CD62L expression, (**C**) CD14, (**D**) CD16 expression, (**E**) CD54 expression, (**F**) CD66b expression and (**G**) CD64 expression. Note, because CD62L for one of the donors expressed multiple peaks in the fresh cells, we excluded it from the fresh and frozen comparison. Phenotype data were analysed using FlowJo_v10.10.0_CL, and graphs were created in GraphPad Prism Version 10.1.2 (324). See Supplementary Material for further flow cytometry analysis and testing of additional donors.

Using the multi-colour panel, we evaluated the labelling of surface receptors using cryopreserved neutrophils compared to fresh neutrophils. Samples were analysed in technical replicates at identical voltages for the fluorochrome, and receptor expression was quantified using MFI (Figure 6, and Supplementary Material Figure S2). Overall, labelling of all receptors was detected. Labelling of CD62L, CD66b and CD64 showed little or no difference among cryopreserved versus fresh neutrophils, whereas there was lower labelling of CD14 and CD16 and higher labelling of CD32a among cryopreserved neutrophils. CD14 expression was high in both fresh and cryopreserved cells in the multi-colour panel, whereas the corresponding CD14 expression in single-stain controls was close to the compensation baseline (unstained autofluorescence).

Overall, our findings suggest that the neutrophil cryopreservation and thawing method preserves detectable the surface receptor expression necessary for neutrophil immunophenotyping. Although there were differences between cryopreserved and fresh neutrophils, the differences did not impact the neutrophil immunophenotype outcomes.

## 4. Discussion

We successfully developed and characterized neutrophil immunophenotypes preserved using our novel cryopreservation method. Our flow analysis revealed that the cryopreserved neutrophils could be stained, and the surface receptors distinguished. The surface receptors detected on fresh neutrophils, including FcγRs (CD16, CD32a, CD64) and CD54, CD62L, CD66b and CD14 were detectable on cryopreserved neutrophils. Differences were observed in the FSC-A versus SSC-A profiles as well as the staining intensity of certain receptors between cryopreserved and fresh neutrophils, which may reflect the technical effects associated with cryopreservation, fixation, or both, known to cause changes in cell structure and receptor conformation [30].

Since neutrophils are among the first immune cell responders during an infection, profiling their phenotypes and responses is crucial in understanding pathogenesis and immunity. Neutrophils have a short lifespan ex vivo [35], thereby requiring immediate investigation. This presents practical challenges in clinical research as assays can be laborious and time-consuming, meaning that only small sample sizes can be investigated. In addition, studies in settings with limited laboratory facilities or other resource constraints may require samples to be shipped to a suitable laboratory for analysis of immune responses using neutrophil profiling. Therefore, developing a neutrophil cryopreservation method that can preserve cell structure and phenotypes would greatly facilitate investigating neutrophil phenotypes in diverse clinical studies and populations.

Using our novel neutrophil cryopreservation method, we were able to identify neutrophils by gating on CD16 and CD66b co-expression. Interestingly, there was a loss of a small CD16^dim^CD66b^dim^ population after cryopreservation. Alterations in neutrophil size and granularity may increase overlap between dim and bright populations, making the dim subset appear absent [30]. Alternatively, loss of the dim population may reflect epitope masking due to receptor conformational changes causing reduced immunofluorescent detectability [34]. Additionally, the absence of dim cells in the cryopreserved cell population may suggest a preferential loss of subsets during freezing and thawing [36,37,38]. All target receptors of interest (FcγRs, CD54, CD62L, and CD66b) were detectable on cryopreserved neutrophils. However, the frequencies of cells expressing CD64 and CD14 differed between cryopreserved and fresh cells. There are other reports that suggest that cryopreservation can impact cell frequencies [38]. Further investigation to determine the effects of cryopreservation on neutrophil subpopulations may be warranted in future studies, depending on the cellular phenotypes being investigated.

Non-activated neutrophils under homeostatic conditions are thought to express low surface levels of CD64 and CD14, with the majority residing intracellularly [39,40,41]. Consistent with the literature, low CD64 labelling was observed across the experiments.

In multi-colour panels, CD14 labelling was higher with fresh than cryopreserved neutrophils. Therefore, further investigation is needed to determine the cause of the increased signal. The staining intensity of receptor CD16 was reduced, whereas CD32a was increased among cryopreserved neutrophils. These variations in receptors may suggest cryopreserved-associated immunophenotypic modifications and need to be considered in future studies of neutrophils in clinical settings such as for infection and inflammation. Further investigation is needed to determine whether these changes are associated with altered neutrophil function. Despite the differences between fresh and cryopreserved neutrophils, our findings support using cryopreserved cells for phenotyping studies of neutrophils.

While all the surface receptors we studied were detected on neutrophils, other relevant surface molecules should be assessed in future studies, including applications where rare or infrequent cell phenotypes are being studied. Furthermore, future studies investigating the functional activities of cryopreserved neutrophils would be valuable. Given the observed differences in surface molecule labelling between fresh and cryopreserved neutrophils, studies involving phenotypic comparisons between subjects or groups should ensure that neutrophil samples are processed and stored consistently.

## 5. Conclusions

In summary, we developed a cryopreservation and thawing method that demonstrates the feasibility of detecting neutrophil receptor expression in cryopreserved samples from healthy donors. Future studies using these protocols with clinical samples to investigate neutrophil phenotypes in specific infections or inflammation will be valuable.

## Figures and Tables

**Table 1:** Preserving and thawing conditions tested.

Condition No.	Cryopreserving Buffer	Thawing Buffer	Thawing Buffer Temp.	Re-Suspension Buffer	Proportion of Cells with Compromised Integrity
1 **	10% BSA + 7.5%DMSO + RPMI	10% BSA, 1XHBSS	4 °C	10% BSA, 1XHBSS	99% population cells had intact membranes **
2	10% DMSO + 90% FBS	50% FBS heat inactivated (HI), RPMI	4 °C	10% FBS (HI), RPMI	Over 95% cells had compromised membrane integrity
3	10% DMSO + 90% FBS	50% FBS (HI), RPMI	37 °C	10% FBS (HI), RPMI	Over 95% cells had compromised membrane integrity
4	10% DMSO + 90% FBS	500 mL RPMI, 50 mL FBS (HI), 5 mL (200 mM) L-Glutamine, 8.4 mL HEPES, 1 mL Gentamicin, 250 µL Minocycline (2 mg/mL) (R10-GM medium)	4 °C	R10-GM medium	Over 90% cells had compromised membrane integrity
5	10% DMSO + 90% FBS	- R10-GM medium	37 °C	R10-GM medium	Over 90% of cells had compromised membrane integrity
6	10% DMSO + 90% FBS	1XPBS (Mg^2+^ and Ca^2+^)^-^, 50% FBS (HI), 2 mM EDTA	4 °C	1XPBS (Mg^2+^ and Ca^2+^)^-^, 1% FBS (HI), 2 mM EDTA	About 70% of the cells showed loss of membrane integrity after one hour
7	10% DMSO + 90% FBS	1XPBS (Mg^2+^ and Ca^2+^)^-^, 50% FBS (HI) + 2 mM EDTA, 2 mg/mL Lignocaine	4 °C	1XPBS (Mg^2+^ and Ca^2+^)^-^, 1% FBS (HI), 2 mM EDTA	Over 50% of the cells had compromised membrane integrity
8	10% DMSO + 90% FBS	1XHBSS	4 °C	1XHBSS	About 50% of cells had compromised membrane integrity
9	10% DMSO + 90% FBS	1XPBS (Mg^2+^ and Ca^2+^)^-^, 50% FBS (HI), 50 mM EDTA	4 °C	1XPBS (Mg^2+^ and Ca^2+^)^-^, 1% FBS (HI), 1 mM EDTA	Over 50% of the cells showed compromised membrane integrity
10	10% DMSO + 90% FBS	1XHBSS, RPMI	4 °C	1XHBSS, RPMI	Approximately 40% of cells had compromised membrane integrity
11	10% DMSO + 90% FBS	1% BSA, 1XHBSS	4 °C	1% BSA, 1XHBSS	Approximately 40% of cells had compromised membrane integrity
12	10% DMSO + 90% FBS	10% BSA, 1XHBSS	4 °C	10% BSA, 1XHBSS	Over 50% of cells had compromised membrane integrity
13	10% DMSO + 90% FBS	10% BSA, 1XHBSS, RPMI	4 °C	10% BSA, 1XHBSS, RPMI	Over 50% of cells had compromised membrane integrity
14	10% BSA + 7.5%DMSO + RPMI	1XHBSS	4 °C	1XHBSS	Approximately 30% cells had compromised membrane integrity
15	10% BSA + 7.5%DMSO + RPMI	1XHBSS, RPMI	4 °C	1XHBSS, RPMI	Approximately 30% proportion of cells had compromised membrane integrity
16	10% BSA + 7.5%DMSO + RPMI	1% BSA, 1XHBSS	4 °C	1% BSA, 1XHBSS	Over 40% cells had compromised membrane integrity
17	10% BSA + 7.5%DMSO + RPMI	10% BSA, 1XHBSS, RPMI	4 °C	10% BSA, 1XHBSS + RPMI	Over 10% proportion of cells had compromised membrane integrity

** Represents optimal working conditions. Cell viability was assessed using Trypan blue exclusion, which stains dead cells.

## Data Availability

Data generated in this study are available from the authors on reasonable request. All reagents used in this study are commercially available.

## Supplementary Materials

The following supporting information can be downloaded at: https://www.mdpi.com/article/10.3390/biology15211951/s1, Figure S1: Neutrophils after thawing using different conditions; Table S1: Antibody master mix (fresh neutrophils); Table S2: Antibody master mix (cryopreserved neutrophils); Figure S2: Examples of cell surface staining of fresh and cryopreserved neutrophils. Two different donors are shown using fresh neutrophils (A and B) and cryopreserved (C and D).
