# Supplementary material for: A Novel Method for Cryopreservation of Human Neutrophils for Phenotypic Analysis
Source: Biology (Basel). 2026 Oct 30;15(21):1951. doi: 10.3390/biology15211951 (PMC13761910; doi:10.3390/biology15211951)
Supplement: Supplementary file 1 [file biology-15-01951-s001.zip › biology-4575345-supplementary.pdf]

Supplementary

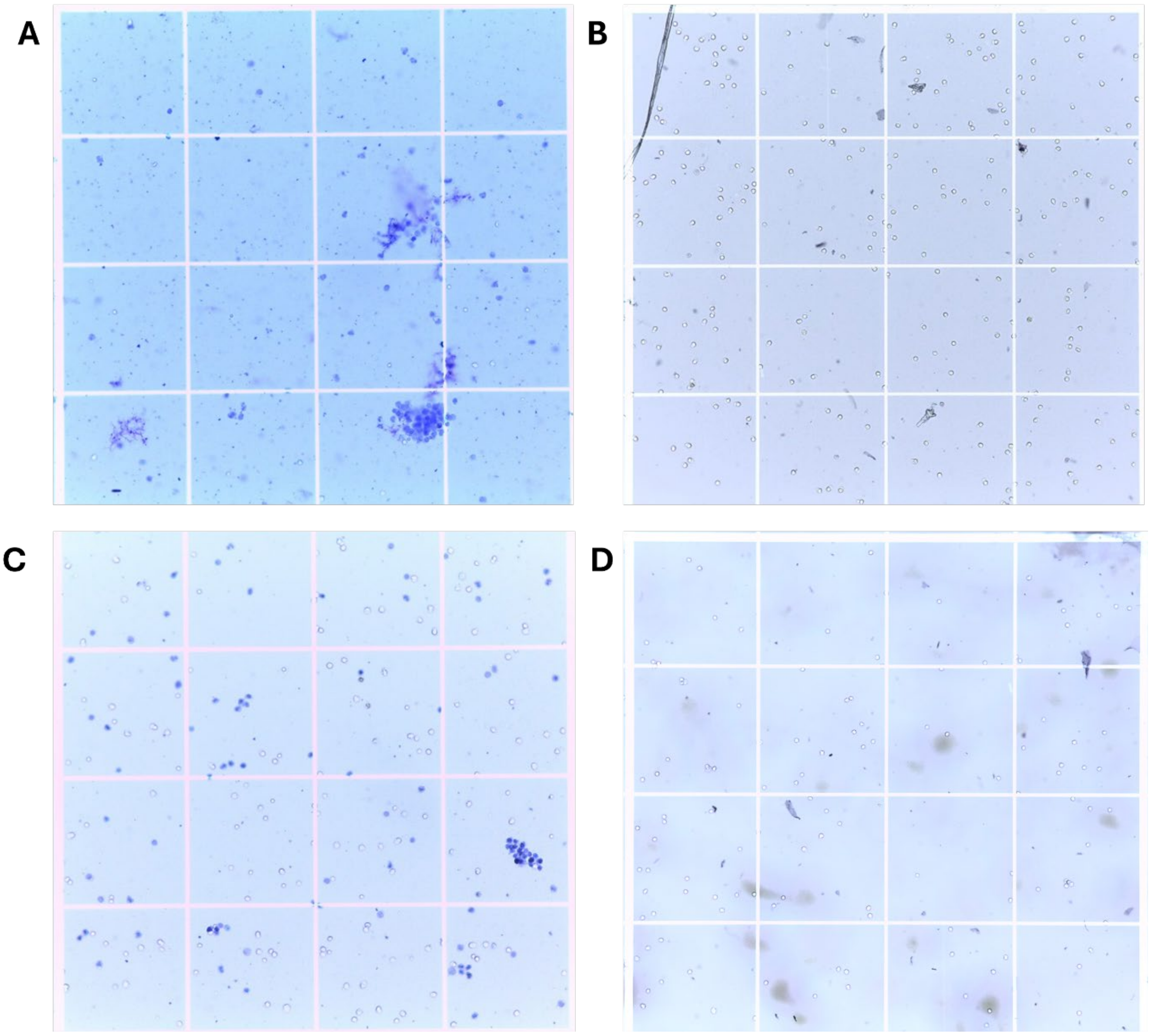

Figure S1. Neutrophils after thawing using different conditions.

A) Neutrophils cryopreserved in 10% DMSO and 90% FBS and thawed with 50% FBS in RPMI-1640. The cells were subsequently filtered using a Falcon 70-µm nylon cell strainer (Corning™ 352350) and resuspended in 10% FBS RPMI. **B)** Neutrophils cryopreserved in 10% BSA and 7.5% DMSO in RPMI-1640, thawed and resuspended in 10% BSA in 1XHBSS (**optimal condition**). **C)** Neutrophils cryopreserved in 10% BSA and 7.5% DMSO in RPMI-1640, thawed and resuspended in 1% BSA in 1XHBSS. **D)** Freshly isolated neutrophils. Images show a corner of the Hemocytometer grid, with each small square measuring 50-µm x 50-µm. Samples were stained

with Trypan Blue exclusion dye which stains dead cells. The images were taken at X10 magnification. Images were captured using LAS EZ Version 3.3.0 (Copyright © 2016, Leica Microsystems Limited, Switzerland). This image is a representation of 7 experiments and multiple donors (5).

Protocol

Materials

Reagents

- Peripheral blood (whole blood)
- StemCell Technologies EasySep™ Direct Human Neutrophil Isolation Kit
- 10 x PBS [-] calcium and [-] Magnesium
- Roswell Park Memorial Institute (RPMI)-1640 medium
- 10 x Dulbecco's Phosphate Buffered Saline (PBS)
- 10 x Hanks' Balanced Salt Solution (HBSS)
- Fetal Bovine Serum (FBS)
- Dimethyl sulfoxide (DMSO)
- Bovine serum albumin (BSA) powder
- 2% FBS-PBS StemCell Technologies
- Ethylenediaminetetraacetic acid calcium disodium salt hydrate (EDTA) (Sigma-Aldrich, Cat. No. ED2SC)
- FACS Buffer (1 x HBSS containing 2% FBS and 0.04% Sodium azide)
- 20% Paraformaldehyde (PFA)

- Trypan Blue Stain (0.4%)
- HEPES buffer (4-(2-hydroxyethyl)-1-piperazineethaesulfonic acid)
- CD66b, PerCP/Cyanine5.5 anti-human, clone G10F5, isotype Mouse IgM, κ, Cat # 305107, BioLegend (San Diego, USA)
- CD14, Brilliant Violet 750™ anti-human, clone 63D3, isotype Mouse IgG1, κ, Cat # 367136, BioLegend (San Diego, USA)
- CD16, Brilliant Violet 421™ anti-human, clone 3G8, isotype Mouse BALB/c x DBA/2, Cat # 562874, BD Horizon (Franklin Lakes, USA)
- CD62L, Alexa Fluor® 488 anti-human, clone DREG-56, isotype Mouse IgG1, κ, Cat # 304816, BioLegend (San Diego, USA)
- CD11b, Brilliant Violet 570™ anti-human, clone ICRF44, isotype Mouse IgG1, κ, Cat # 301325, BioLegend (San Diego, USA)
- CD64, APC anti-human, clone 10.1, isotype Mouse IgG1, κ, Cat # 305014, BioLegend (San Diego, USA)
- CD32, PE/Cyanine7 anti-human, clone FUN-2, isotype Mouse IgG2b, κ, Cat # 303214, BioLegend (San Diego, USA)
- CD54, PE anti-human, clone HA58, isotype Mouse IgG1, κ, Cat # 353106, BioLegend (San Diego, USA)

Equipment

- Lithium heparin vacutainers (BD Vacutainer LH 170 I.U (REF 367526))
- EasyEights™ EasySep™ Magnet
- Nunc CryoTube 1.8 mL vials (Thermo Scientific; Cat. 368632)
- Corning CoolCell LX Cell Freezing Containers

- 2 mL Cryopreservation tubes (cryovials)
- 70-µm nylon strainer
- Eppendorf tubes
- Haemocytometer and cover slip
- Light Microscope
- Pipette and pipette tips
- Vortex
- 14 mL Polystyrene tubes (FACS tubes)
- 50 mL Falcon tube
- 10 mL Serological pipettes
- 25 ml Serological pipettes
- 1000 µL pipette tips
- Pipette guns
- 96 well U-bottom plates
- Timer
- BD LSR II flow cytometer

Reagents setup

- R10-GM buffer (RPMI 1640 + 10% FBS + L-Glutamine (200 mM) + Penicillin/Streptomycin (100x) + HEPES buffer (1M))
- 10% FBS in RPMI 1640
- 10% BSA in 1x HBSS
- FACS Buffer (1% BSA and 0.04% sodium azide in 1 x PBS)

- BD CompBead Anti-Mouse Ig, κ/Negative Control Particles Set catalogue no. BDB552843
- 2% PFA in 1 x PBS

SOP

Neutrophil isolation

1. Collect peripheral blood in a 10 mL lithium heparin tube BD Vacutainer LH 170 I.U (REF 367526).
2. Transfer 5 mL of whole blood into a 50 mL Falcon tube.
3. Add 4 times i.e., 20 mL of 2% FBS in 1 x PBS) (Cat. No. 07905 StemCell Technologies) to the 5 mL whole blood.
4. Gently mix the whole blood and 2% FBS in 1 x PBS with a pipette and subsequently centrifuge at 300 x g, room temperature, 5 minutes.
5. Vacuum aspirating the supernatant.
6. Add EDTA at a final concentration of 1 mM.
7. Transfer the blood into a 14 mL polystyrene tube.
8. Vortex RapidSpheres for 30 seconds.
9. Add 250 µL (50 µL/mL) Isolation cocktail and 250 µL RapidSpheres to the whole blood and incubated at room temperature for 5 minutes.
10. Add 7 mL of 2% FBS in 1 x PBS and mix gently.
11. Transfer the uncapped tube onto the EasyEight magnet and incubate at room temperature for 10 minutes.
12. Gently transfer the enriched cell suspension into a new polystyrene tube.

13. Add 250 µL RapidSpheres to the enriched cells and mix gently.
14. Incubate at room temperature for 5 minutes on a tube rack.
15. Transfer the tube onto the magnet for isolation.
16. Transfer the enriched cells into a new polystyrene tube and place the tube on the magnet for a final separation.
17. Aspirate the enriched cells and filter through a 70-µm nylon strainer into 50 mL falcon tubes.
18. Centrifuge the cells for 300 x g, room temperature, 5 minutes.
19. Vacuum aspirate the supernatant.
20. Resuspend the cells in 2 mL RPMI-1640 enriched with 10% FBS, L-Glutamine (200 mM), Penicillin/Streptomycin (100 x), 20 mM HEPES buffer and 1/160 heat-inactivated human serum and keep the sample on ice.
21. Count the cells with the hemocytometer to determine the concentration of the cells.
22. To cryopreserve the neutrophils, centrifuge the cells (300 x g, 4°C, 5 minutes).
23. Vacuum aspirate the supernatant and ensure to remove as much liquid as possible.
24. Resuspend the cells in medium containing 7.5% DMSO and 10% BSA in RPMI-1640 at 0.1 - 1 x 10^7 neutrophils/mL d store at -80 °C.
25. Transfer 1 mL of the cells into cryovials and place the cryovials into Corning CoolCell alcohol-free freezing container(s).
26. Transfer the CoolCell alcohol-free freezing container(s) into -80 °C freezer (for long term storage, transfer the frozen neutrophils into liquid nitrogen).

Staining fresh neutrophils

27. Transfer 50 µL of neutrophil cells at concentration $2x10^6$ cells/mL into the 96-well plate.

28. Centrifuge the cells for 300 x g at room temperature for 4 minutes.
29. Discard the supernatant.
30. Add 25 µL of master mix of staining antibodies for 30 minutes on ice in the dark (Table S1).

Table S1: Antibody master mix (fresh neutrophils)

Fluorophore	Marker	1x Volume
PerCP/Cyanine5.5 anti-human	CD66b	1.25
Brilliant Violet 750™ anti-human	CD14	1.25
Brilliant Violet 421™ anti-human	CD16	0.31
Alexa Fluor® 488 anti-human	CD62L	0.31
APC anti-human	CD64	0.63
PE/Cyanine7 anti-human	CD32a	0.63
PE anti-human	CD54	0.31
Brilliant Violet 570™ anti-human*	CD11b	0.63
	Buffer	19.68
	**Total**	**25 µL**

31. Add 100 µL FACS buffer enriched with 1% (w/v) BSA and 0.04% (w/v) sodium azide in in 1x PBS buffer to the cells and centrifuge the plate for 300 x g at 4°C for 4 minutes.
32. Discard the supernatant.
33. Wash twice with 150 µL FACS buffer for 300 x g at 4°C for 4 minutes.
34. Discard the supernatant.
35. Add 50 µL of 2% PFA fixative and incubate for 15 minutes in the dark on ice.
36. Add 100 µL FACS buffer to stop the reaction.
37. Centrifuge the cells for 300 x g at 4°C for 4 minutes.
38. Discard the supernatant.
39. Add 150 µL FACS buffer and store at 4 °C in the dark overnight before acquisition.
40. Acquire cells by flow cytometry (using BD LSR II).

Staining compensation beads for multicolour flow cytometry

41. Transfer 2 mL FACS buffer to a 10 mL Falcon tube.
42. Vortex both negative and positive beads for 30 seconds.
43. Add two drops of negative beads to the FACS buffer.
44. Add two drops of positive beads to the FACS buffer.
45. Vortex the beads for 30 seconds to mix.
46. Centrifuge the beads at 1500 rpm at 4°C for 6 minutes.
47. Vacuum aspirate the supernatant.
48. Resuspend the beads in 2200 µL.
49. Transfer 100 µL beads to well labelled Eppendorf tubes according to the antibody stains needed (also include unstained beads).
50. Add antibodies to the beads at final concentration 1x.
51. Incubate beads for 25 minutes on ice in the dark, with gentle mixing (by tapping the tubes) at 5 minutes and 15 minutes mark.
52. Vortex the beads and add 800 µL of FACS buffer to each Eppendorf tube, vortex again, and centrifuge at 1500 rpm at 4°C for 6 minutes.
53. Vacuum aspirate the supernatant and leave ~ 30 µL in the tube.
54. Add 50 µL of PFA at final concentration 2% and incubate for 15 minutes in the dark on ice.
55. Add 800 µL of FACS buffer, vortex, and centrifuge at 1500 rpm at 4°C for 6 minutes.
56. Vacuum aspirate the supernatant and leave ~ 30 µL in the tube.
57. Add 200 µL of FACS buffer and vortex to resuspend the beads.

Staining cryopreserved neutrophils

58. Remove cryopreserved neutrophils from the -80 °C freezer or liquid nitrogen and keep the samples on dry ice.

59. Thaw cryopreserved neutrophils in a water bath at 37 °C by agitating the tube until a small piece of ice remains (ensure the tube cap is above water).
60. Wipe the tube with 80% ethanol saturated paper towel.
61. Transfer the neutrophils into 9 mL of 10% BSA in 1x HBSS buffer in a 15 mL Falcon tube.
62. Mix gently by inverting three times.
63. Centrifuge the cells at 200 x g at 4°C for 10 minutes.
64. Aspirate supernatant and resuspend cells in 1 mL 10% BSA in 1x HBSS.
65. Add 1 mL of 4% PFA and incubated for 15 minutes in the dark at 4 °C.
66. Add 6 ml FACS buffer.
67. Centrifuge the cells at 300 x g at 4°C for 4 minutes.
68. Aspirate supernatant and leave ~ 100 µl of supernatant to resuspend.
69. Transfer 50 µl of cells into a 96-well round bottom plate.
70. Centrifuge the cells at 300 x g at 4°C for 4 minutes.
71. Discard the supernatant.
72. Add 25 µl of antibody master mix and incubate for 30 minutes at 4 °C in the Dark (Table S2).

Table S2: Antibody master mix (cryopreserved neutrophils)

**Fluorophore**	**Marker**	**1x Volume**
PerCP/Cyanine5.5 anti-human	CD66b	1.25
Brilliant Violet 750™ anti-human	CD14	1.25
Brilliant Violet 421™ anti-human	CD16	0.31
Alexa Fluor® 488 anti-human	CD62L	1.25
APC anti-human	CD64	0.63
PE/Cyanine7 anti-human	CD32a	0.63
PE anti-human	CD54	0.31
	Buffer	19.68
	**Total**	**25 µl**

73. Add 125 µL of FACS buffer and centrifuge at 300 x g at 4°C for 4 minutes.

74. Discard the supernatant.

75. Add 150 μL of FACS buffer and centrifuge at 300 x g at 4°C for 4 minutes.

76. Discard the supernatant.

77. Add 200 μL of FACS buffer and transfer the cells to FACS tubes.

78. Store at 4 °C until acquisition on the flow cytometer (within 24 hours).

Figure S2. Examples of cell surface staining of fresh and cryopreserved neutrophils. Two different donors are shown using fresh neutrophils (A and B) and cryopreserved (C and D)

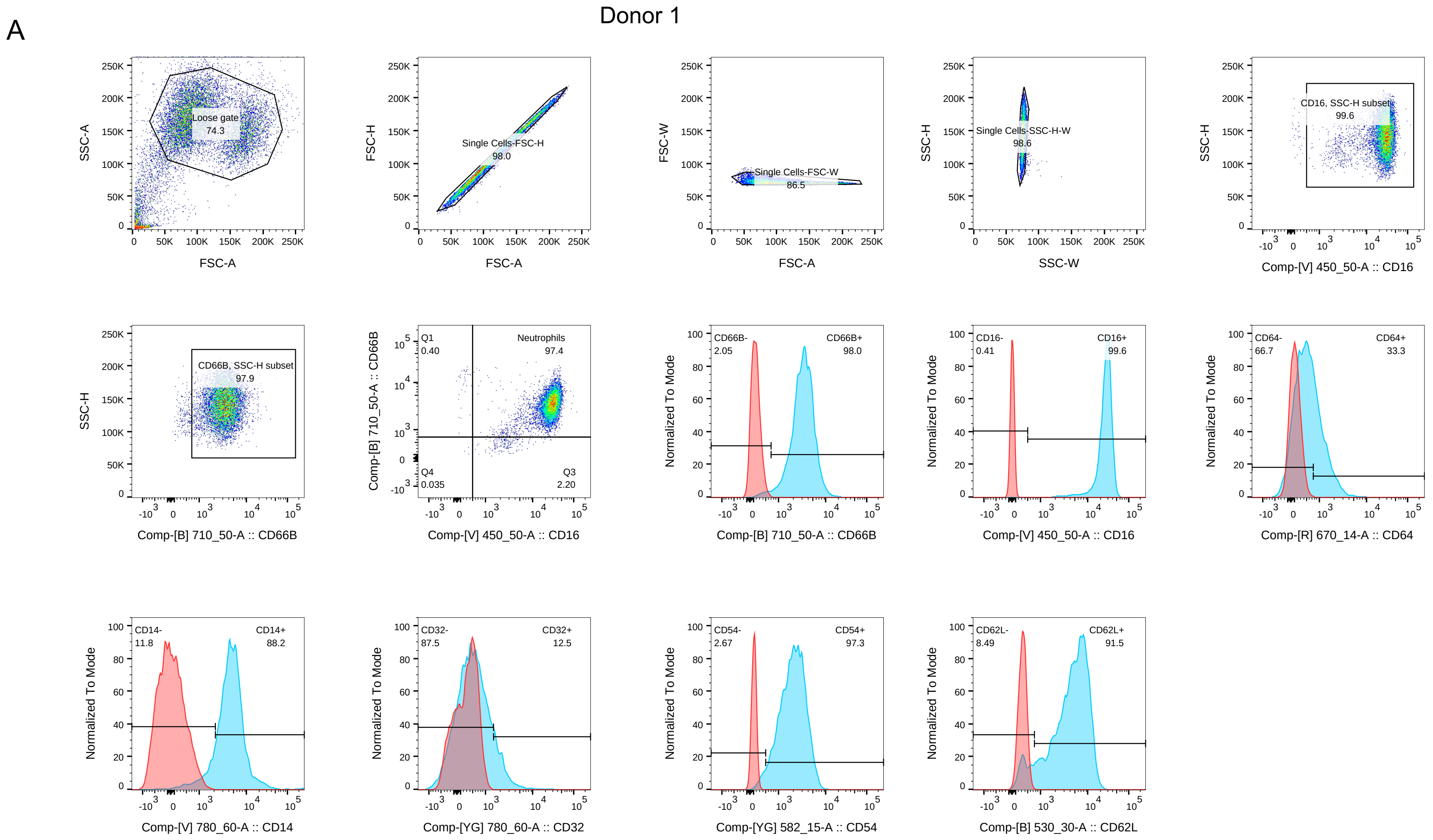

B

Donor 2

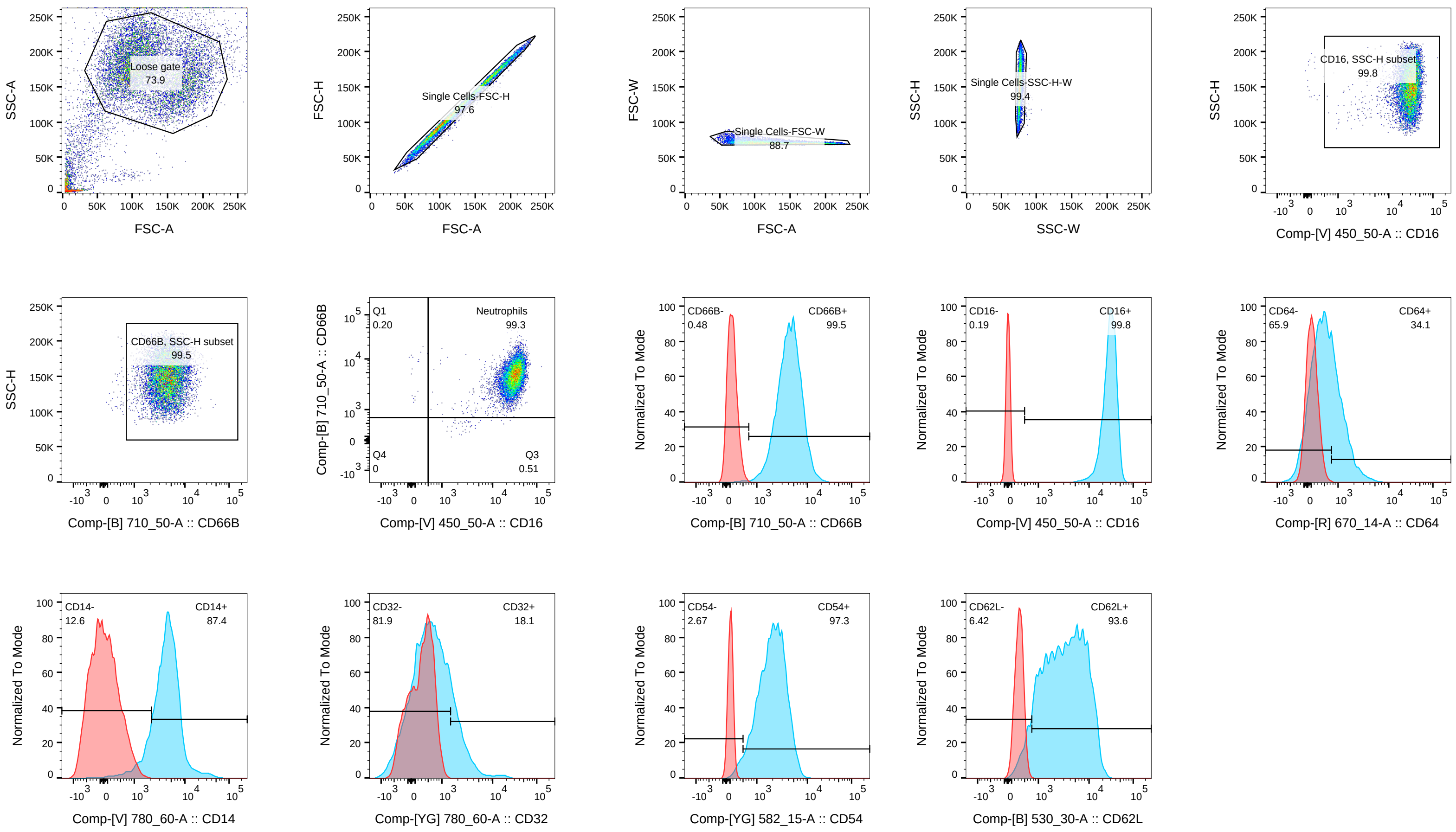

Loose gate
73.9
SSC-A
FSC-A
Single Cells-FSC-H
97.6
FSC-H
FSC-A
Single Cells-FSC-W
88.7
FSC-W
FSC-A
Single Cells-SSC-H-W
99.4
SSC-H
SSC-W
CD16, SSC-H subset
99.8
SSC-H
Comp-[V] 450_50-A :: CD16
CD66B, SSC-H subset
99.5
SSC-H
Comp-[B] 710_50-A :: CD66B
Q1
0.20
Neutrophils
99.3
Q4
0
Q3
0.51
Comp-[B] 710_50-A :: CD66B
Comp-[V] 450_50-A :: CD16
CD66B-
0.48
CD66B+
99.5
Normalized To Mode
Comp-[B] 710_50-A :: CD66B
CD16-
0.19
CD16+
99.8
Normalized To Mode
Comp-[V] 450_50-A :: CD16
CD64-
65.9
CD64+
34.1
Normalized To Mode
Comp-[R] 670_14-A :: CD64
CD14-
12.6
CD14+
87.4
Normalized To Mode
Comp-[V] 780_60-A :: CD14
CD32-
81.9
CD32+
18.1
Normalized To Mode
Comp-[YG] 780_60-A :: CD32
CD54-
2.67
CD54+
97.3
Normalized To Mode
Comp-[YG] 582_15-A :: CD54
CD62L-
6.42
CD62L+
93.6
Normalized To Mode
Comp-[B] 530_30-A :: CD62L

Donor 1

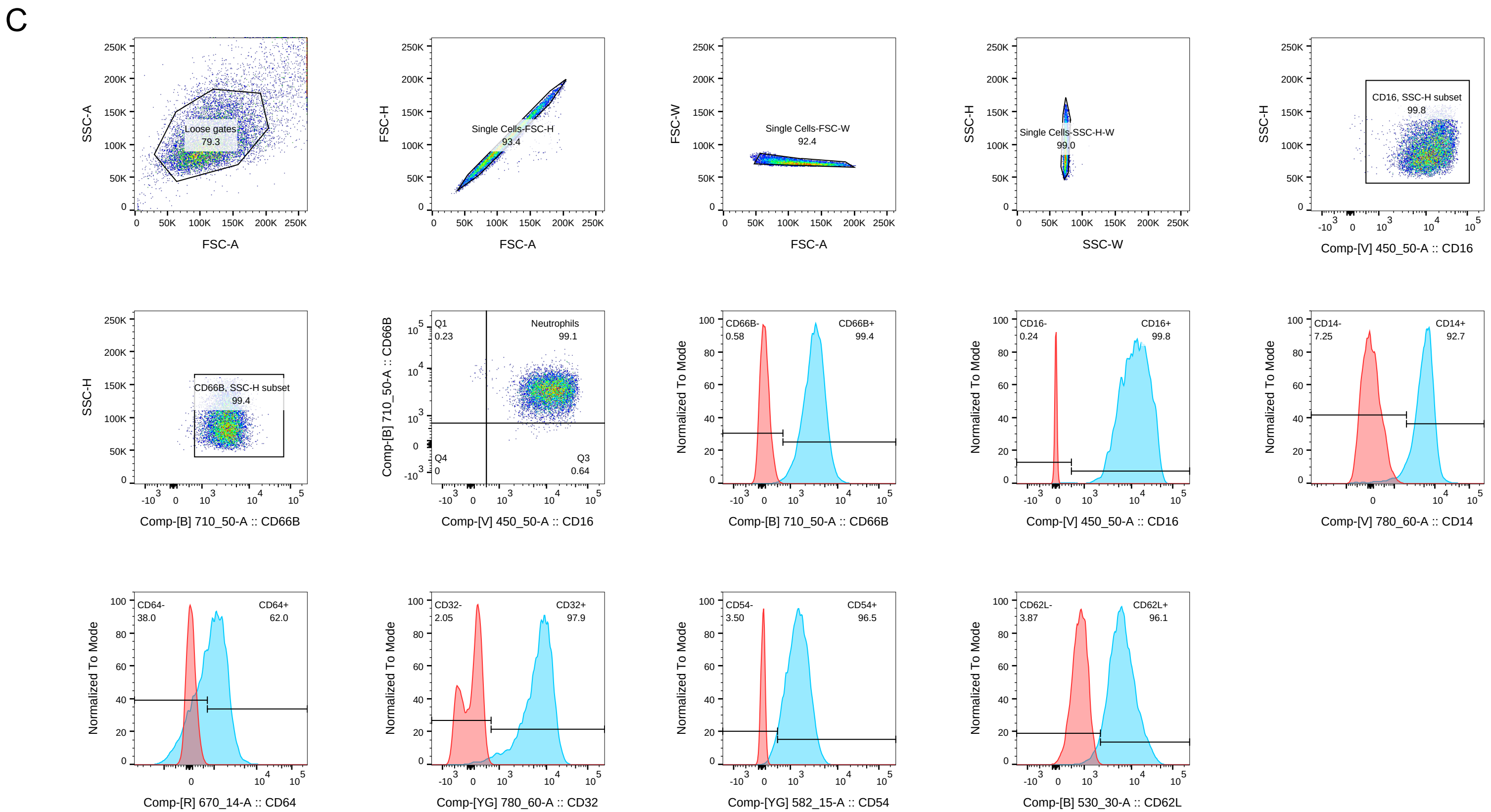
C
Loose gates
79.3
SSC-A
FSC-A
Single Cells-FSC-H
93.4
FSC-H
FSC-A
Single Cells-FSC-W
92.4
FSC-W
FSC-A
Single Cells-SSC-H-W
99.0
SSC-H
SSC-W
CD16, SSC-H subset
99.8
SSC-H
Comp-[V] 450_50-A :: CD16
CD66B, SSC-H subset
99.4
SSC-H
Comp-[B] 710_50-A :: CD66B
Q1
0.23
Neutrophils
99.1
Q4
0
Q3
0.64
Comp-[B] 710_50-A :: CD66B
Comp-[V] 450_50-A :: CD16
CD66B-
0.58
CD66B+
99.4
Normalized To Mode
Comp-[B] 710_50-A :: CD66B
CD16-
0.24
CD16+
99.8
Comp-[V] 450_50-A :: CD16
CD14-
7.25
CD14+
92.7
Comp-[V] 780_60-A :: CD14
CD64-
38.0
CD64+
62.0
Comp-[R] 670_14-A :: CD64
CD32-
2.05
CD32+
97.9
Comp-[YG] 780_60-A :: CD32
CD54-
3.50
CD54+
96.5
Comp-[YG] 582_15-A :: CD54
CD62L-
3.87
CD62L+
96.1
Comp-[B] 530_30-A :: CD62L

D

Donor 2

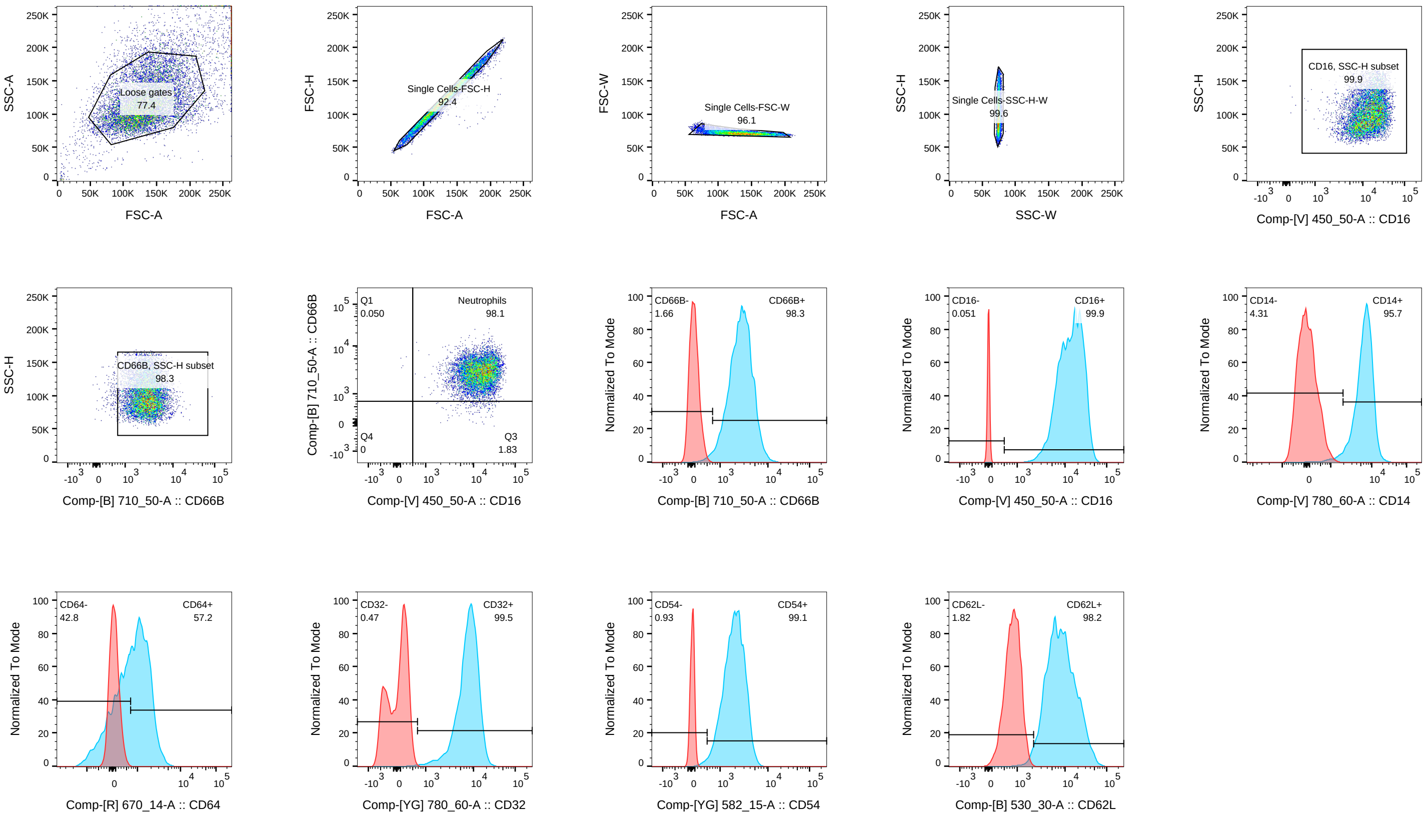
Loose gates
77.4
SSC-A
FSC-A
Single Cells-FSC-H
92.4
FSC-H
FSC-A
Single Cells-FSC-W
96.1
FSC-W
FSC-A
Single Cells-SSC-H-W
99.6
SSC-H
SSC-W
CD16, SSC-H subset
99.9
SSC-H
Comp-[V] 450_50-A :: CD16
CD66B, SSC-H subset
98.3
SSC-H
Comp-[B] 710_50-A :: CD66B
Q1
0.050
Neutrophils
98.1
Q4
0
Q3
1.83
Comp-[B] 710_50-A :: CD66B
Comp-[V] 450_50-A :: CD16
CD66B-
1.66
CD66B+
98.3
Normalized To Mode
Comp-[B] 710_50-A :: CD66B
CD16-
0.051
CD16+
99.9
Normalized To Mode
Comp-[V] 450_50-A :: CD16
CD14-
4.31
CD14+
95.7
Normalized To Mode
Comp-[V] 780_60-A :: CD14
CD64-
42.8
CD64+
57.2
Normalized To Mode
Comp-[R] 670_14-A :: CD64
CD32-
0.47
CD32+
99.5
Normalized To Mode
Comp-[YG] 780_60-A :: CD32
CD54-
0.93
CD54+
99.1
Normalized To Mode
Comp-[YG] 582_15-A :: CD54
CD62L-
1.82
CD62L+
98.2
Normalized To Mode
Comp-[B] 530_30-A :: CD62L
